# Open-Source Syringe Pump Library

**DOI:** 10.1371/journal.pone.0107216

**Published:** 2014-09-17

**Authors:** Bas Wijnen, Emily J. Hunt, Gerald C. Anzalone, Joshua M. Pearce

**Affiliations:** 1 Department of Materials Science & Engineering, Michigan Technological University, Houghton, Michigan, United States of America; 2 Department of Electrical & Computer Engineering, Michigan Technological University, Houghton, Michigan, United States of America; Imperial College London, United Kingdom

## Abstract

This article explores a new open-source method for developing and manufacturing high-quality scientific equipment suitable for use in virtually any laboratory. A syringe pump was designed using freely available open-source computer aided design (CAD) software and manufactured using an open-source RepRap 3-D printer and readily available parts. The design, bill of materials and assembly instructions are globally available to anyone wishing to use them. Details are provided covering the use of the CAD software and the RepRap 3-D printer. The use of an open-source Rasberry Pi computer as a wireless control device is also illustrated. Performance of the syringe pump was assessed and the methods used for assessment are detailed. The cost of the entire system, including the controller and web-based control interface, is on the order of 5% or less than one would expect to pay for a commercial syringe pump having similar performance. The design should suit the needs of a given research activity requiring a syringe pump including carefully controlled dosing of reagents, pharmaceuticals, and delivery of viscous 3-D printer media among other applications.

## Introduction

Free and open source (or libre) technological development is a fundamentally new, decentralized, participatory and transparent system to create both software and hardware. It stands in sharp contrast to the closed box, top-down, and secretive standard commercial approach to development [1]. As much of the Internet now relies on free and open-source software (FOSS), open source is becoming the norm in software development [2],[3]. FOSS has been so successful that for many applications it is the defacto standard, with 94% of the World’s top 500 supercomputers, 75% of the top 10,000 websites and 98% of enterprises using open-source software [4],[5]. FOSS is computer software made available as source code (open source) that can be used, studied, copied, modified, and redistributed without restriction, or with restrictions that only ensure that further recipients have the same rights under which it was obtained [6]. FOSS is in widespread use in science and engineering and has driven down the cost of numerical simulation in a number of fields ranging from psychotherapy [7] and medicine [8],[9], neural circuit reconstruction [10], genomic sequences annotation [11], education [12],[13], and ecology [14]. In addition, it has been proposed as a solution to the intellectual property tragedy in nanotechnology, which has slowed progress and deployment in the field [15],[16],[17],[18]. Even greater cost reductions for science, however, can be found with the application of open source hardware [19],[20],[21],[22]. The development of open-source hardware has the potential to radically reduce the cost of performing experimental science and put high-quality scientific tools in the hands of everyone from the most prestigious labs to rural clinics in the developing world [19],[22],[23].

This article introduces a low-cost open-source family of syringe pumps. Creation of parametric open-source designs using an open-source computer aided design (CAD) package is described to produce customized syringe pumps for scientific and/or health applications. Details are provided for use of open-source RepRap 3-D printers to fabricate the components. An open-source Rasberry Pi computer used as a wireless control device is also illustrated. The performance of the pumps produced is assessed and the method’s advantages, known limitations and potential for radically reducing the cost of doing science are discussed.

## Materials and Methods

The low-cost open-source family of syringe pumps are completely customizable allowing both the volume and the motor to scale for specific applications. The bill of materials for the three variations of the syringe pump are shown in Table S1. The user/designer must first determine the size of motor to enable that application. The appropriate motor size can be selected once the required torque is known following [24]. A bigger motor provides more torque, but necessitates larger printed components. A bigger syringe allows more fluid to be pushed out, both per second and in total, but decreases the precision of the device. A simple change to the OpenSCAD script specifying the motor selection defines the dimensions for the printed parts.

### OpenSCAD and Open-source 3-D Printing

Open-source and freely available OpenSCAD is script-based, parametric CAD software possessing powerful 3-D modeling capabilities [25]. It is not graphical; models are created by adding and subtracting primitives to produce the desired shape. It supports creation and extrusion of polygons and poly lines, so can be used to create very complex shapes. The script language is based upon C++ and only a few methods are required to produce very complex designs, so the learning curve is short, albeit steep for those not possessing programming experience. The scripts are written such that designs are parametric – the design can easily be altered by changing key dimensions. For instance, the syringe pump script can be altered to produce parts fitting different motors simply by specifying which motor to design for. The script written for the syringe pump is available online [26]. Models rendered in OpenSCAD are typically exported as stereolithography (stl) files for the first step in producing a 3-D print using any of the RepRap 3-D printers currently available. Images of syringe pump parts rendered by OpenSCAD and photographs of the printed parts are shown in Figures S1–S10 in File S1.

RepRap printers almost universally require g-code, a human-readable file format specifying the path the print head must follow to produce a physical object from a software model. G-code is produced by software referred to as a “slicer”, which, as the name implies, slices an stl model into layers each having the same thickness in the z-direction. Cura was used to slice the syringe pump stl models [27]. Cura is also open-source and freely available.

The parts were printed with RepRap3-D printers. Two different printer designs, a Cartesian [28] and a delta printer [29], were used to produce the parts out of 1.75 mm polylactic acid (PLA) filament. The printer design employed is ultimately irrelevant as both produce shapes using exactly the same method and materials and are different only in the way the print head is moved. Both printers were equipped with hot ends having 0.5 mm nozzles and prints were sliced at a layer height of 0.25 mm and a print speed of 60 mm/s. Parts were printed in plates, that is all of the printed parts needed to assemble a syringe pump were printed in one printer cycle.

RepRap printers typically interface with a host program running on a computer but can also run independently, reading g-code stored on a memory card. The syringe pump was printed using a host computer running ReptierHost [30] another freely available, open-source software written specifically for RepRap and RepRap-like 3D printers. For detailed instruction on the construction and operation of the printers see [28], [29].

### Syringe Pump Control and Interface

**Figure 1:**
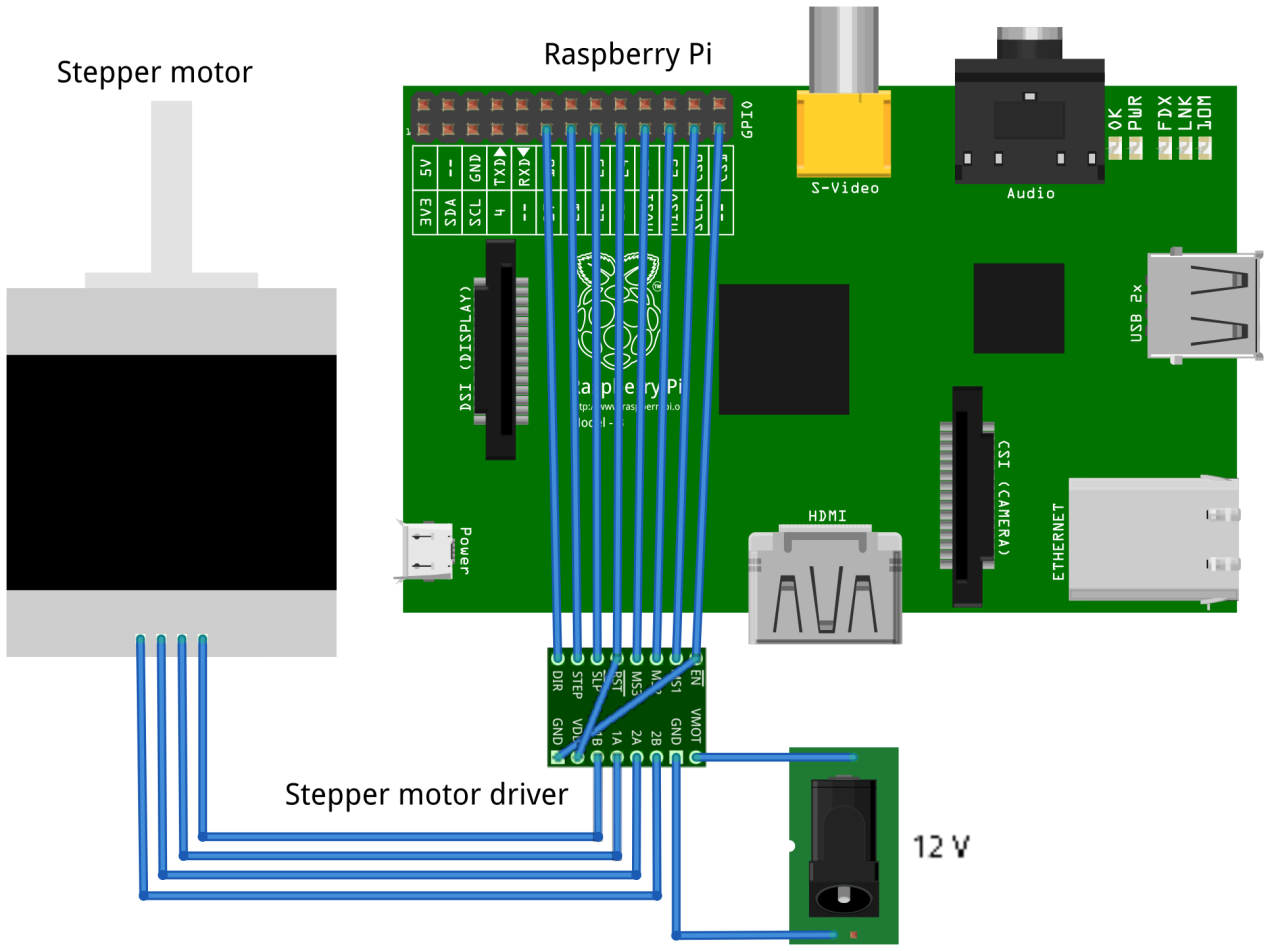
Electronic schematic of Open-Source Syringe Pump.

**Figure 2:**
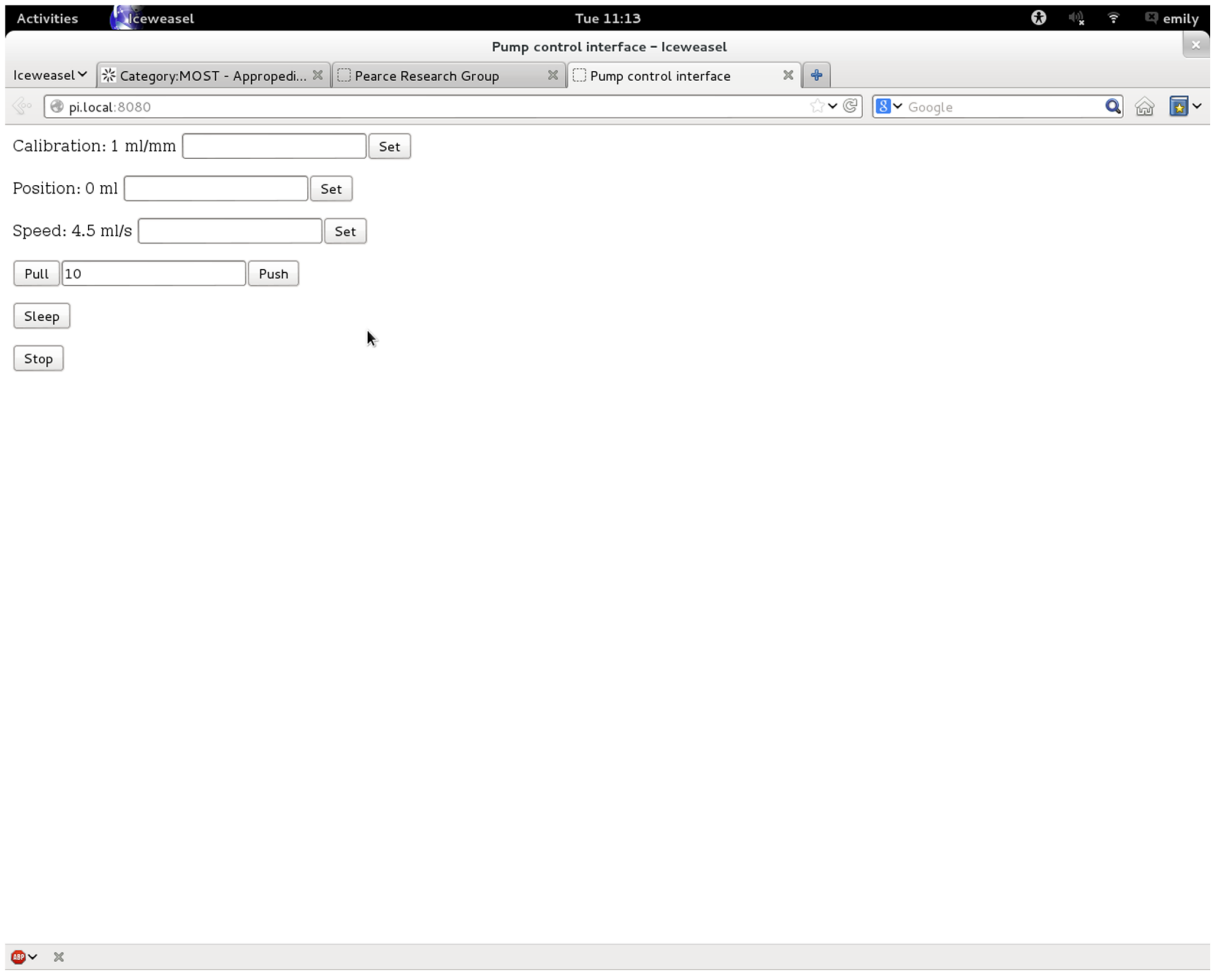
Screenshot of Syringe Pump Web Interface.

The syringe pump is controlled by an open-source Python program developed here [26] running on a Raspberry Pi, which is an ARM based computer running GNU/Linux [31], [32]. The Raspberry Pi is an inexpensive, credit card-sized computer having integrated networking, sound, video, USB host and most importantly, exposed and readily accessible I/O lines. The wiring diagram for the syringe pump controller (Figure 1) utilizes a single Pololu A4988 stepper controller, which controls the stepper motor that drives the syringe pump. The Raspberry Pi is installed with the standard Raspbian operating system [32]. A custom web server is run, which serves a web page via either wired network or wirelessly via a wireless USB adapter attached to the Raspberry Pi’s USB port. Any computer on the network can then control the pump through this web page (Figure 2).

### Calibration and Performance Assessment Methodology

The pump is calibrated by setting it up with an initial calibration value set to 1 mL/mm. A small arbitrary volume appropriate for the size of syringe used is pushed twice from the syringe and the actual value of the second push is measured. This is done to partially account for drops staying on the end of the syringe. This number is divided by the amount the syringe was told to push out, the resulting number goes into the calibration window. The sequence is repeated three times to ensure correct calibration.

The force produced by the lead-screw actuated design was measured by placing the assembled syringe pump with a steel rod in place of the syringe in a frame along with with a 30 kg-capacity scale. The pump was oriented such that the motor end sat upon the scale and the steel plunger faced upward, pressing against a fixed platform. The pump motor was advanced until it stalled or a component failed and the maximum force produced was read off the scale display.

The pumps’ maximum delivery rate is a function of the speed at which the motor stalls. Stall speed was determined by increasing pulse rate to the motor until it stalled and then decreasing to the point where it ran again, establishing the maximum speed and therefore maximum delivery rate.

Precision was tested by repeated delivery of a preset volume (fixed by setting the total number of motor steps) of distilled water onto a Mettler AE100 scale having a readability of 0.1 mg. The relative humidity within the weighing chamber was maintained in a saturated state by placing containers of distilled water in it, permitting it to equilibrate and then ensuring that it was kept well sealed for the duration of the assessment. Performance of both the NEMA11 and NEMA17 pumps was assessed at different microstepping settings.

## Results

**Figure 3:**
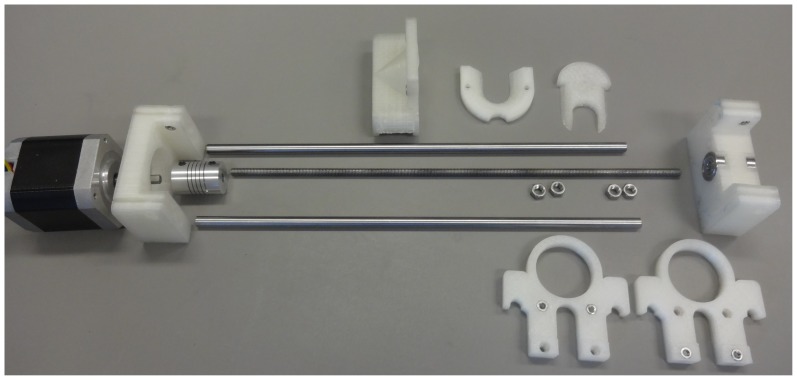
Exploded view of Open-Source Syringe Pump.

**Figure 4:**
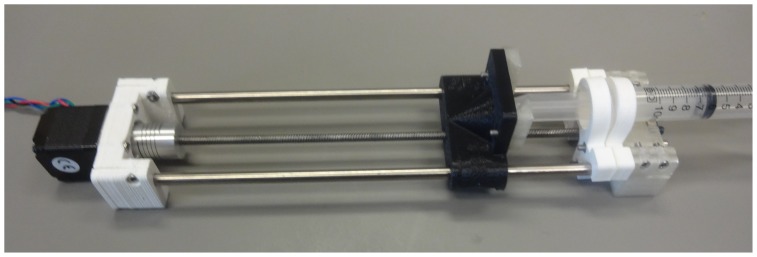
Digital Photograph of Open-Source Syringe Pump version Nema 11.

**Figure 5:**
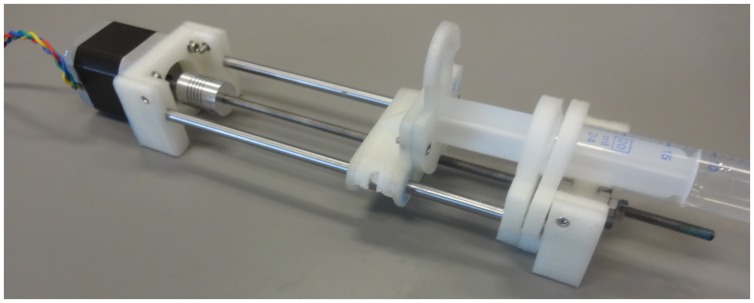
Digital Photograph of Open-Source Syringe Pump version Nema 17.

**Figure 6:**
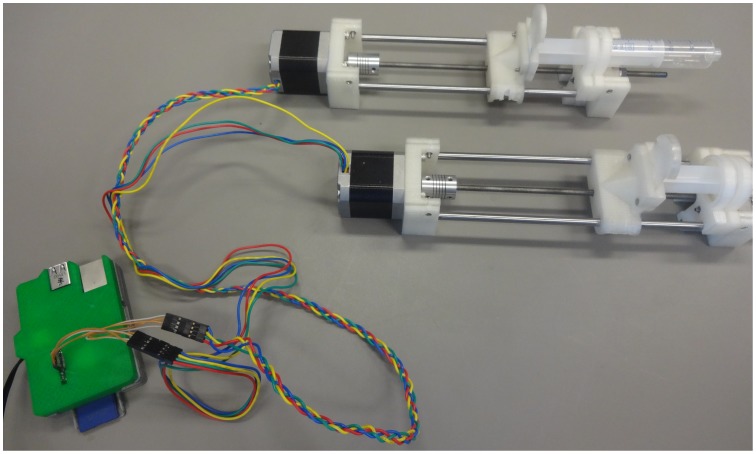
Digital Photograph of Open-Source Syringe Pump version of the Dual Nema 17 Pump.

**Figure 7:**
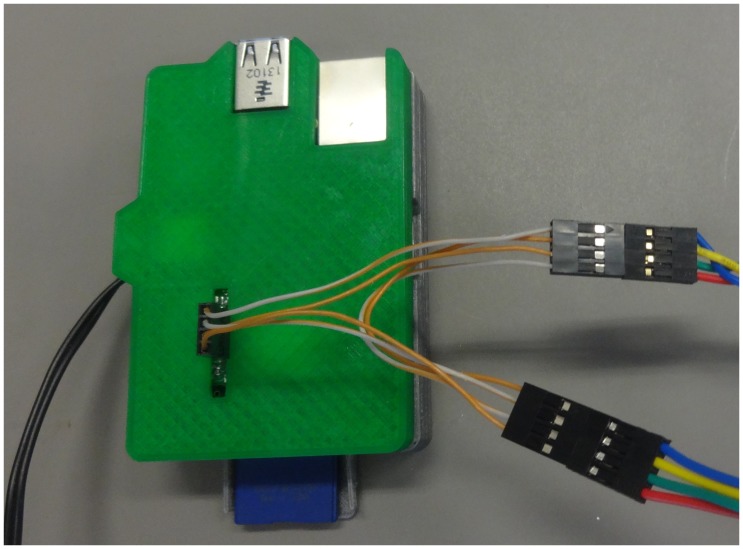
Digital Photograph of the Dual Pump connection.

Three different pumps were assembled, all of which are relatively easy to construct from the parts shown in exploded view in Figure 3. Assembled pumps are shown in Figures 4, 5 and 6 for the Nema 11, Nema 17 and dual Nema 17 pumps, respectively. The dual version consists of two identically sized pumps connected in parallel to the motor controller (Figure 7). The pumps are driven synchronously at the same rate. The controller has the capacity to drive more than one pump simultaneously if required. If one of the connections from the connector to the pump shown in Figure 7 is reversed then the two pumps will go in opposite directions.

The force developed by the OS syringe pump depends on the motor used. When pushing on an immovable object, the NEMA17 version produced 200 N, and the NEMA11 version produced 93 N. Force did not appear to be affected by the microstepping rate, probably due to the nature of lead-screw actuation that is inefficient at translating force into rotation, particularly with the thread pitch and profile used in this design. The force developed was sufficient to cause damage to some of the printed parts, none of which resulted in impairment and new parts could quickly be printed and replaced.

When calibrated with a 25 mL syringe, the NEMA17 version yielded a maximum delivery rate of 2.1 mL/s and the NEMA11 version yielded 1.4 mL/s when calibrated with 10 mL syringe. Accuracy was +/−1% for the NEMA11 and +/−5% for the NEMA17 measured in 1 mL increments. Precision was found to be relatively insensitive to microstepping for both the NEMA11 and NEMA17 (Table 1). The coefficient of variation when delivering approximately 1 mL of distilled water was about 3% or less regardless of microstepping and it is very likely that precision is actually better than reported as the measurement method was limited to the volume of a single drop (e.g. ∼20 micro Liters). It is unlikely that microstepping need be employed as the 200 step/revolution motors coupled with a properly sized syringe should provide virtually any resolution demanded.

**Table 1:** Coefficient of variation as a function of microstepping for NEMA 11 and NEMA 17 open-source syringe pumps.

Coefficient of variation			
	Microstepping		
	1	4	16
**NEMA11**	1.17%	0.56%	1.55%
**NEMA17**	3.66%	2.13%	2.26%

It is clear that using open-source methods reduced the cost of the pumps considerably from commercial pumps as summarized in Table 2. The single syringe pumps have a part cost under $100 using hardware from online retailers. This includes the Raspberry Pi controller that permits control of the syringe pump from virtually every web enabled device available. Commercial syringe pumps can cost anywhere from $260 to over $5000 as seen in Table 2.

**Table 2:** Specifications for the open-source syringe pump are shown compared to commercial pumps.

Name	Price	Description	Speed	Force	Other
Most OS SyringePump Nema 11	$90.00	Completely customizable, always knowswhere the syringe is, fits any size syringe,failsafe stop button.	Max: 1.4 mL/s(10 mL syringe)	Max: 93 N	Accuracy: ±1%, Reproducibility: ±0.6%
Most OS SyringePump Nema 17	$97.00	Completely customizable, always knowswhere the syringe is, fits any size syringe,failsafe stop button.	Max: 2.1 mL/s(25 mL syringe)	Max: 200N	Accuracy: ±5%, Reproducibility: ±2.1%
Most OS Dual SyringePump Nema 17	$154.00	Completely customizable, holds 2or more syringes,depending on how many connectionsdesired, always knowswhere the syringe is, fits any size syringe,failsafe stop button.	Max: 2.1 mL/s(25 mL syringe)	Max: 200N	Accuracy: ±5%, Reproducibility: ±2.1%
NE-300 “Just Infusion”Syringe Pump	$260	Infusion	Max: 0.417 mL/s	–	Up to 60 mL syringes -infusion ratecan be changed while pumping, INFUSES ONLY
B.Braun/McGawBD 360Syringe Pump	$435	Can be used as primary infusor or can deliverdownstream secondary piggyback infusions.	Max: 0.1 mL/s	–	Delivery Time: 10–60 min. in 2.5 min.increments, Accuracy: ±3%
GenieTouchSyringe Pump	$675	The pump always knows how much is leftin the syringeand how much the handle can be pushed down.Accuracy is based on carraige positiondetection, which is +/−0.2 mm.	Max: 3.68 mL/s	Max: 196.13 N	Auto positioning/stalling detection, Multi-directionalleft or right application
NE-4000 ProgrammableDouble Syringe Pump	$928	Dual pump system allows for continuousinfusion or emulsification.Network, control, and monitor up to100 pumps with one computer.	Max: 2.0 mL/s	Max: 444.82 N(at min speed) or 444.82 N(at max speed)	Dispensing accuracy of +/−1%, Max pumping rate:6120 mL/hr with a B-D 60 cc syringe, Syringeinside diameter range: 0.100 to 50.00 mm
Advanced SyringePump withComputer Controlfrom MedAssociates Inc.	$1343.34	The PHM-111EC is controlled byan internal computer so that exactinfusion rates can be set. Frontpanel switches allow for a simplesetup, thus avoiding complicatedmenus or charts.	Max: 0.119 mL/s(depending on the volumeof the syringe)	Min: 68.95 kPa Max:413.685 kPa dependingon the syringe)	Controls up to 16 pumps
Fusion Touch 400 SyringePump	$1,350	From Chemyx, and can run on selectversions of Windows, Mac and Linux.	Max: 0.167 mL/s(10 mL syringe)	Max: 222.41 N	Step Resolution is 0.016 microns, Accuracy ±0.35%,Reproducibility ±0.05%
Fisher Scientific SingleSyringe Pump	$1,509	Applications: Calibrating, diluting, dispensing,dosing, emulsifying, fluid transfer, infusions	Max: 0.144 mL/s	–	Dispensing Volume: 10 µL to 60 mL, Auto shut offvolume dispense mode
Sono Tek Syringe Pump	$1,800	Accepts up to 2 of the same types of syringes,capacity range from 10 µ to 60 mL. Also has additionalvalving that can set up the pump to fill the syringe.	Max: 0.5 mL/s	–	Single shot and continuous flow operations
Cole-Parmer Dual SyringeInfusion Pump	$2,606	Dual syringe infusion pump	Max: 2.45 mL/s	Max: 275.79 kPa	Accepts 10 µL to 140 mL syringes, Reproducibility ±0.2% -Accuracy±0.5%, Power: 115 VAC
Cole-Parmer ContinuousFlow Syringe Pump	$3,947	Hold up to four syringes to cyclecontinuously back and forth in apush-pull action.As two syringes are infusing,two syringes are withdrawingat the same rate.	Max: 1.17 mL/s	Max: 177.93 N	Syringe size: 4–10 uL to 60 mL, Reproducibility: +/−0.1%,Accuracy: +/− <1%
402 Syringe Pumpfrom Gilson Inc.	$5000–5500	Assures accuracy in sample transfer, dilution,reagent addition, mixing and more.Offers speed and reliability for repetitiveliquid handling tasks.	Max: 2.0 mL/s	Max: 0.8 MPa(0.1–10-mL syringe)to 0.3 MPa(25-mL syringe)	Reproducibility: 0.8% at 10 µL -Injection Volume: 1.0 µL–25 mL,Accuracy: 98.2% at 10 µL

Overall, using completely open source methods, this pump is economical, user friendly, and accurate. Even considering the approximate $500 price of the RepRap 3-D printer, the value of this approach to design and manufacturing far exceeds that of commercial units, particularly for resource starved laboratories.

## Discussion

As has been demonstrated previously, the open-source ecosystem lends itself well to research endeavors, especially with regards to maximizing the value of a research dollar [19],[22],[33],[34]. This is particularly true when designs for desired components, or even designs for similar components to those desired, are made freely available for use and customization [19],[20],[22]. Armed with open-source 3-D printers and hardware, freely available and open-source software and designs, researchers can design and manufacture bespoke apparatus at a small fraction of the price of commercial offerings. The ability to alter and tune designs to produce apparatus that better align with research goals eliminates “making do” with what is available commercially. By way of example, this paper presents an elegantly simple design for a syringe pump that performs admirably and should serve as a good foundation for derivation of better and more useful apparatus for specific research goals.

The simplicity of the design coupled with ready access to its source makes it very easy to customize and construct; even first year students with limited exposure to such activity are able to assemble a complete, working system. The cost of the entire system, including the controller and web-based control interface, is on the order of 5% or less than one would expect to pay for a commercial syringe pump having similar features and performance. The platform is not limited to just use as a syringe pump; it is a relatively high precision linear actuator that can easily be modified for use for positioning, i.e. for stages for microscopy. Similarly it could be used as a head for 3-D printing with viscous media. 3D printing and liquid handling with a syringe pump could be combined as has been done recently by Kitson et al., to produce user-friendly reactionware for chemical synthesis and purification [35]. Using open-source RepRap 3-D printers and the open-source syringe pump developed here chemists not only only have complete control over every aspect of hardware, but can also set up the experiments for a fraction of the cost of commercially available tools.

Incremental improvement of designs in the open-source ecosystem tends to occur organically [22]. It is therefore reasonable to expect that as the population of the interested audience grows, the rate of innovation increases, perhaps at a much greater rate than could be expected in commercial R&D centers. This incremental approach to development not only takes place at a rapid pace, it spreads the cost of development over the entire user/developer community with the currency being predominantly the time spent by the individual developers. Since freely available open-source designs can be made available to the entire globe, even small time investments in development can have significant impact. This is especially important given that the tools developed can be considered appropriate technology and are of particular interest to poorly funded laboratories such as those in undeveloped and developing economies [36]. Development of open-source designs can, from that perspective, be considered a form of philanthropy, although the developer also benefits by the product of his work and the improvements made to it by others [22].

The design presented here is deliberately simple; it is intended to demonstrate the utility and efficiency of the open-source method of development and provide one starting point for derivation of improved designs. There is (by design) ample opportunity for improvement and future work. A syringe pump can be used for a variety of applications requiring carefully controlled dosing of reagents, pharmaceuticals, delivery of viscous 3-D printer media, etc. All of these applications have specific requirements that this endlessly customizable design can be tailored to meet. For instance, microstepping may not be required, making a less expensive motor controller suitable and driving the cost of the syringe pump even lower.

## Conclusions

An open-source and freely available design for a simple to build and customize syringe pump has been provided and working pumps have been constructed and evaluated. The design performs well as compared to much costlier commercial models while permitting virtually endless customization and so should suit the needs of a given research activity requiring a syringe pump. Only readily available, open-source hardware and software were used for the design and manufacture of the pumps, further validating application of open-source methodologies for development of research-ready laboratory equipment.

## Acknowledgments

The authors would like to acknowledge helpful discussions with P. Fraley.

## Supporting Information

File S1 **Figures S1–S10: 3-D printable parts for the open-source syringe pump.** Figure S1 Carriage STL rendering. Figure S2 Carriage digital image. Figure S3 Clamp STL rendering. Figure S4 Clamp digital image. Figure S5 End idler STL rendering. Figure S6 End idler digital image. Figure S7 End motor STL rendering. Figure S8 End motor digital image. Figure S9 Wedges STL rendering. Figure S10 Wedges digital image.
(ZIP)

Table S1 **Bill of Materials for three examples of the open-source syringe pumps.**
(DOC)
