# Supplementary figures and images for: Open-Source Syringe Pump Library
Source: PLoS One. 2014 Sep 17;9(9):e107216. doi: 10.1371/journal.pone.0107216 (PMC4167991; doi:10.1371/journal.pone.0107216)

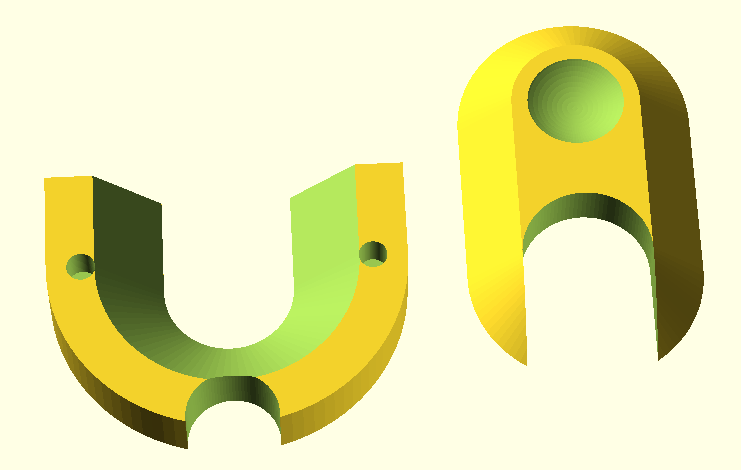

Supplement: File S1 — Figures S1–S10: 3-D printable parts for the open-source syringe pump. Figure S1 Carriage STL rendering. Figure S2 Carriage digital image. Figure S3 Clamp STL rendering. Figure S4 Clamp digital image. Figure S5 End idler STL rendering. Figure S6 End idler digital image. Figure S7 End motor STL rendering. Figure S8 End motor digital image. Figure S9 Wedges STL rendering. Figure S10 Wedges digital image. (ZIP) [file pone.0107216.s001.zip › Figure S10 -Wedges.tiff]

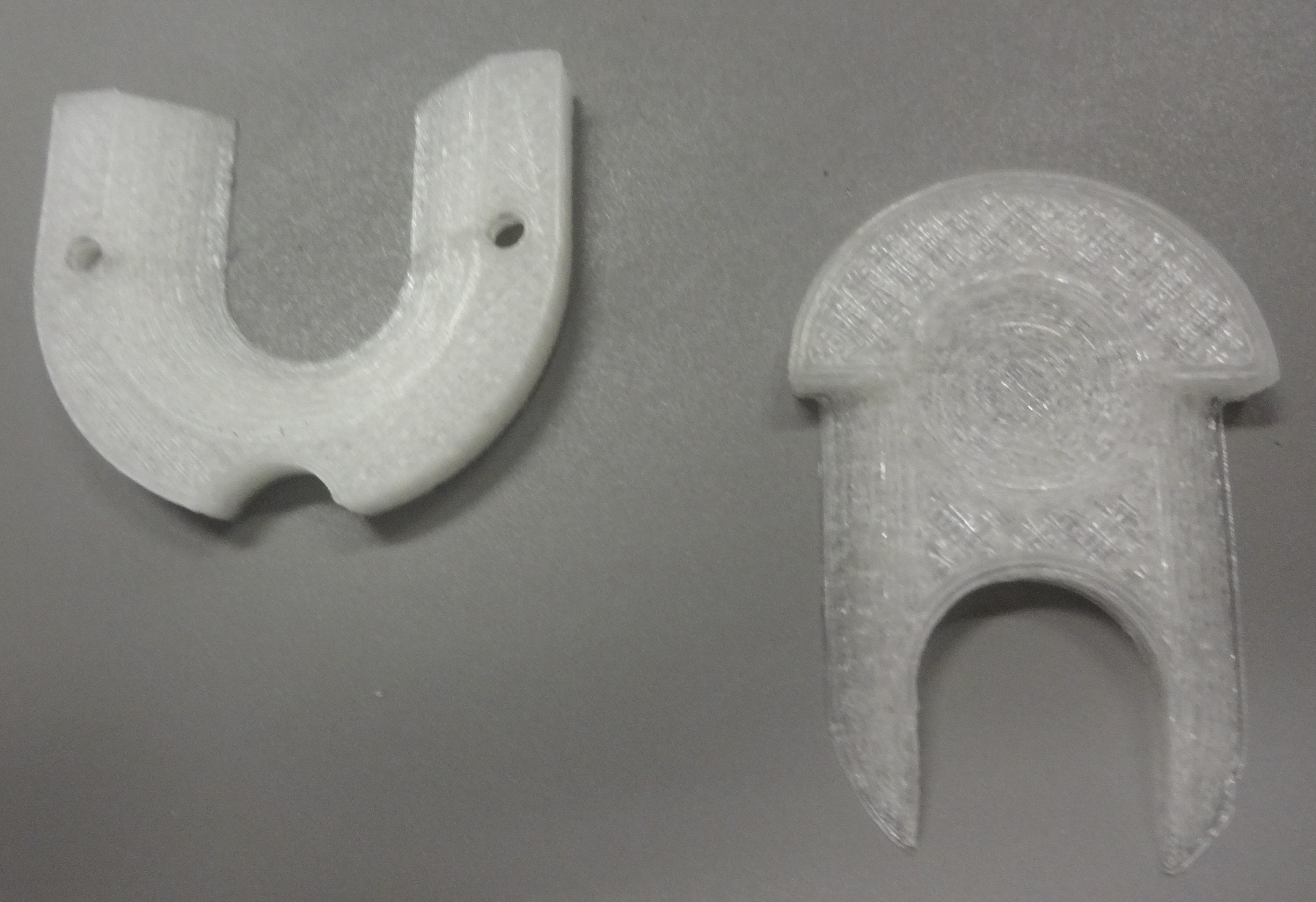

Supplement: File S1 — Figures S1–S10: 3-D printable parts for the open-source syringe pump. Figure S1 Carriage STL rendering. Figure S2 Carriage digital image. Figure S3 Clamp STL rendering. Figure S4 Clamp digital image. Figure S5 End idler STL rendering. Figure S6 End idler digital image. Figure S7 End motor STL rendering. Figure S8 End motor digital image. Figure S9 Wedges STL rendering. Figure S10 Wedges digital image. (ZIP) [file pone.0107216.s001.zip › Figure S9 - Wedge-p.tiff]

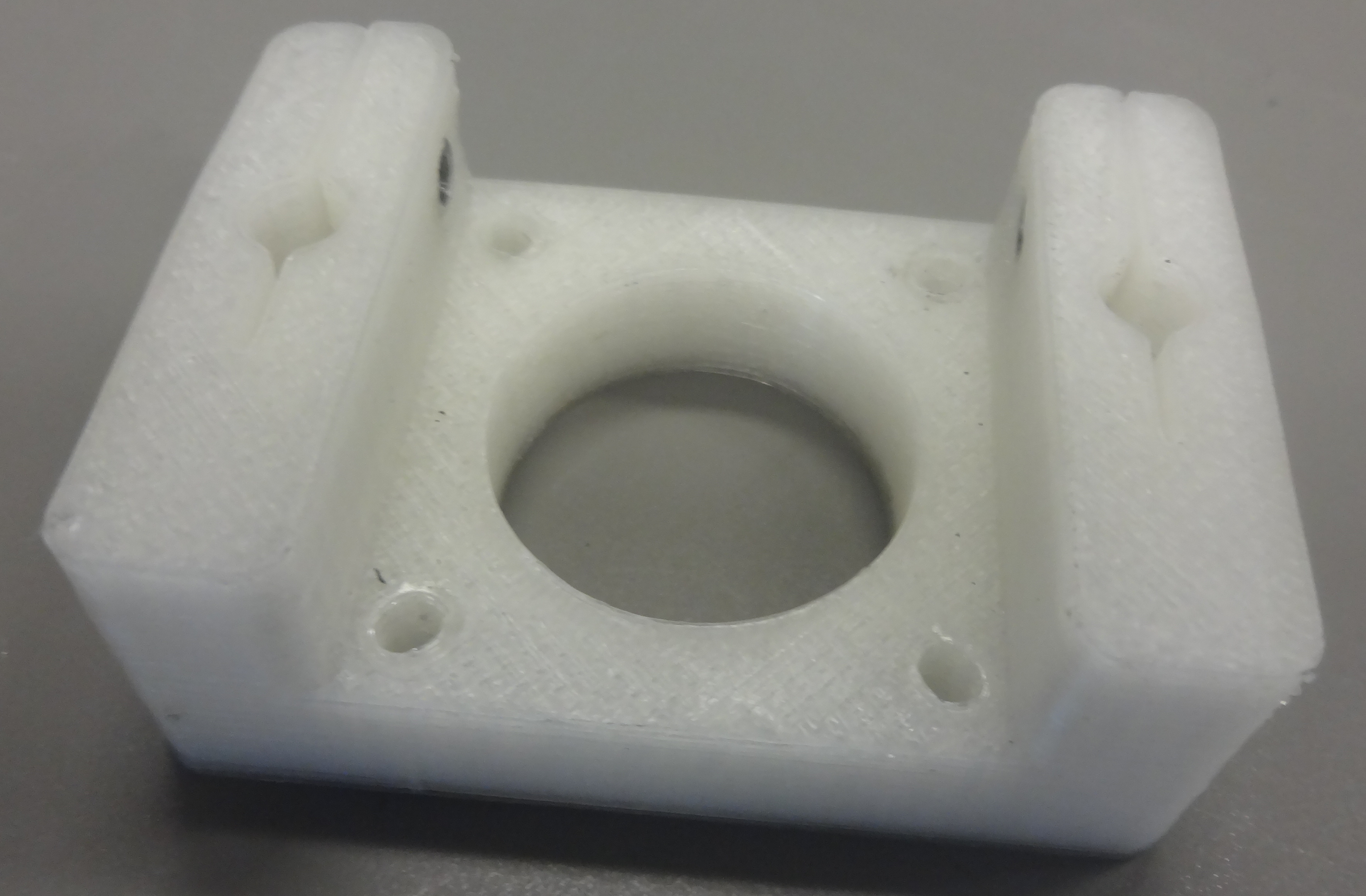

Supplement: File S1 — Figures S1–S10: 3-D printable parts for the open-source syringe pump. Figure S1 Carriage STL rendering. Figure S2 Carriage digital image. Figure S3 Clamp STL rendering. Figure S4 Clamp digital image. Figure S5 End idler STL rendering. Figure S6 End idler digital image. Figure S7 End motor STL rendering. Figure S8 End motor digital image. Figure S9 Wedges STL rendering. Figure S10 Wedges digital image. (ZIP) [file pone.0107216.s001.zip › Figure S8 - EndMotor-p.tiff]

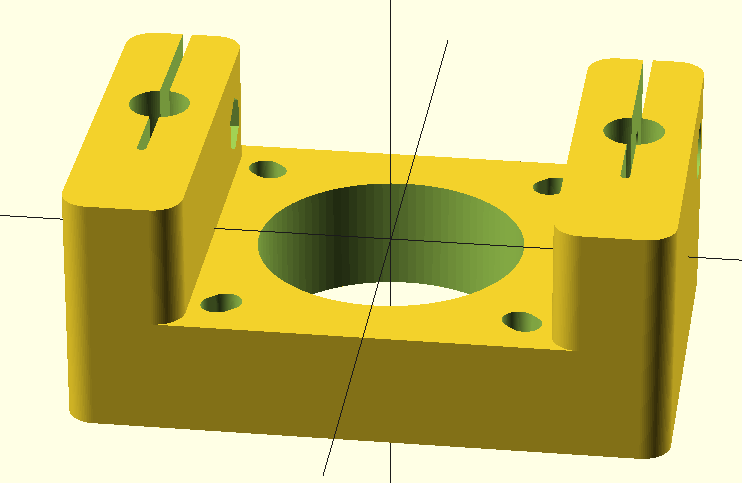

Supplement: File S1 — Figures S1–S10: 3-D printable parts for the open-source syringe pump. Figure S1 Carriage STL rendering. Figure S2 Carriage digital image. Figure S3 Clamp STL rendering. Figure S4 Clamp digital image. Figure S5 End idler STL rendering. Figure S6 End idler digital image. Figure S7 End motor STL rendering. Figure S8 End motor digital image. Figure S9 Wedges STL rendering. Figure S10 Wedges digital image. (ZIP) [file pone.0107216.s001.zip › Figure S7 -EndMotor.tiff]

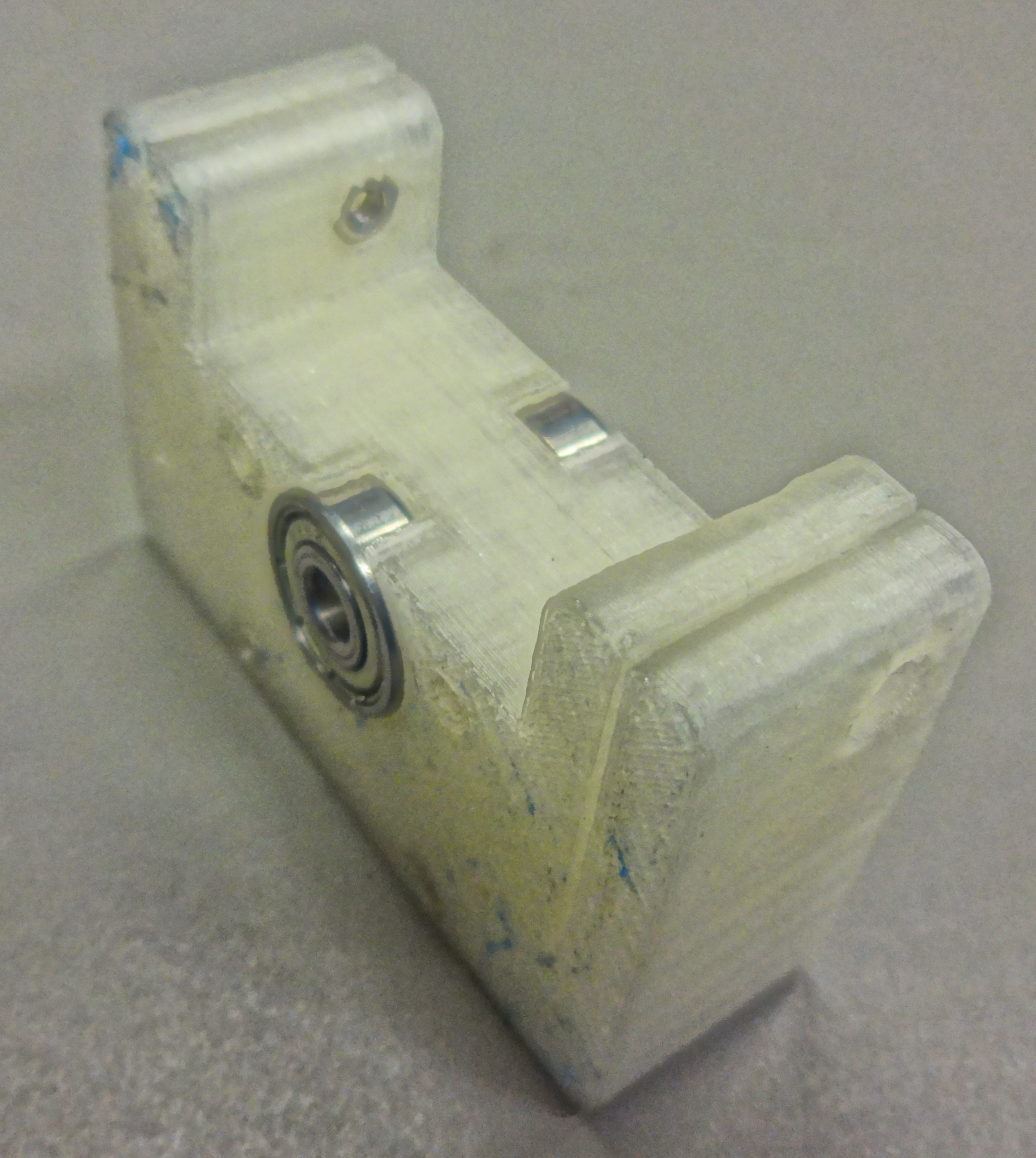

Supplement: File S1 — Figures S1–S10: 3-D printable parts for the open-source syringe pump. Figure S1 Carriage STL rendering. Figure S2 Carriage digital image. Figure S3 Clamp STL rendering. Figure S4 Clamp digital image. Figure S5 End idler STL rendering. Figure S6 End idler digital image. Figure S7 End motor STL rendering. Figure S8 End motor digital image. Figure S9 Wedges STL rendering. Figure S10 Wedges digital image. (ZIP) [file pone.0107216.s001.zip › Figure S6 - EndIdler-p.tiff]

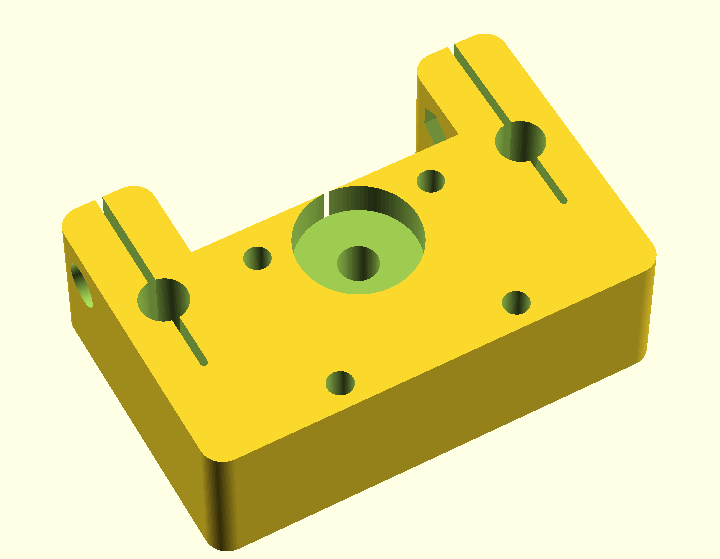

Supplement: File S1 — Figures S1–S10: 3-D printable parts for the open-source syringe pump. Figure S1 Carriage STL rendering. Figure S2 Carriage digital image. Figure S3 Clamp STL rendering. Figure S4 Clamp digital image. Figure S5 End idler STL rendering. Figure S6 End idler digital image. Figure S7 End motor STL rendering. Figure S8 End motor digital image. Figure S9 Wedges STL rendering. Figure S10 Wedges digital image. (ZIP) [file pone.0107216.s001.zip › Figure S5 - EndIdler.tiff]

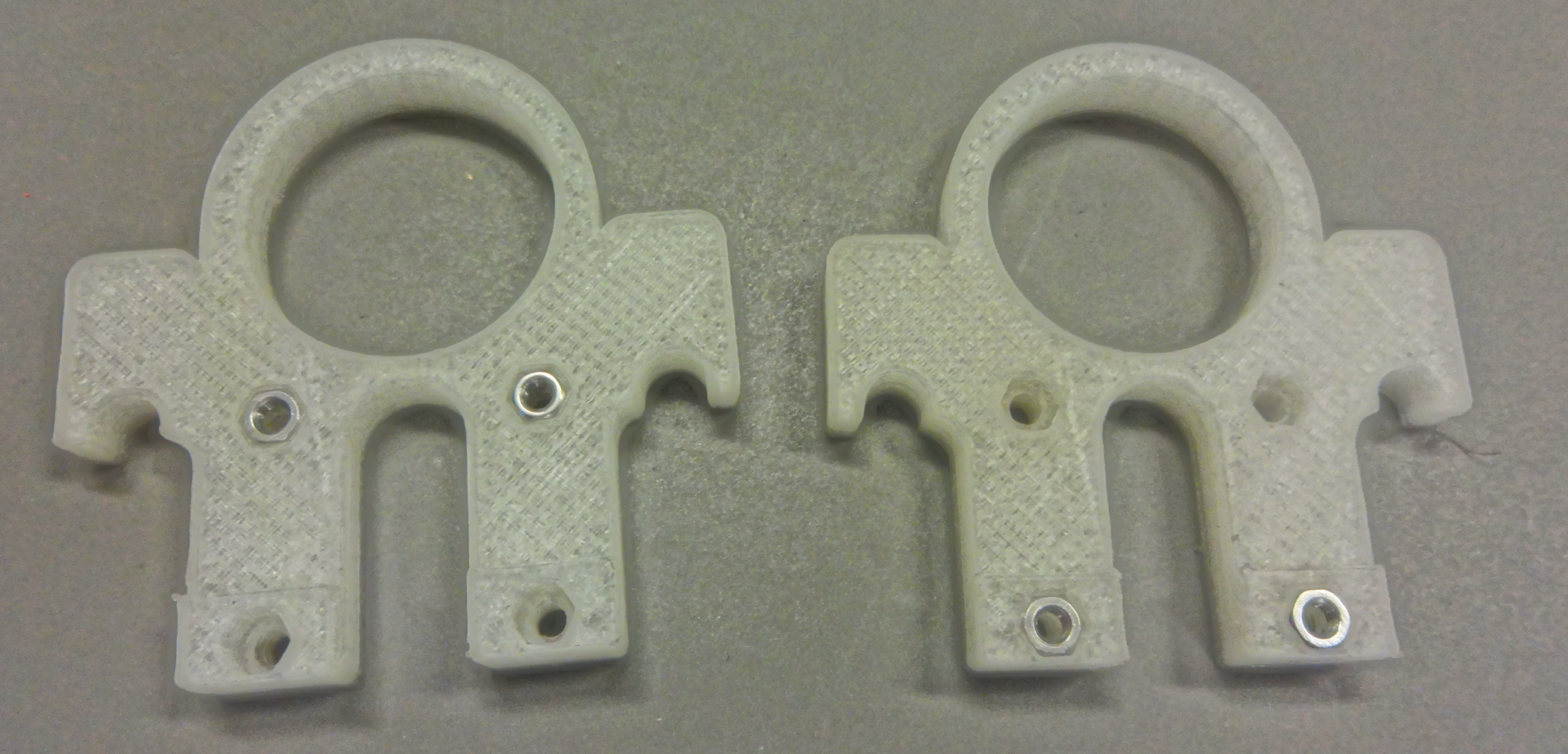

Supplement: File S1 — Figures S1–S10: 3-D printable parts for the open-source syringe pump. Figure S1 Carriage STL rendering. Figure S2 Carriage digital image. Figure S3 Clamp STL rendering. Figure S4 Clamp digital image. Figure S5 End idler STL rendering. Figure S6 End idler digital image. Figure S7 End motor STL rendering. Figure S8 End motor digital image. Figure S9 Wedges STL rendering. Figure S10 Wedges digital image. (ZIP) [file pone.0107216.s001.zip › Figure S4 - Clamp-p.tiff]

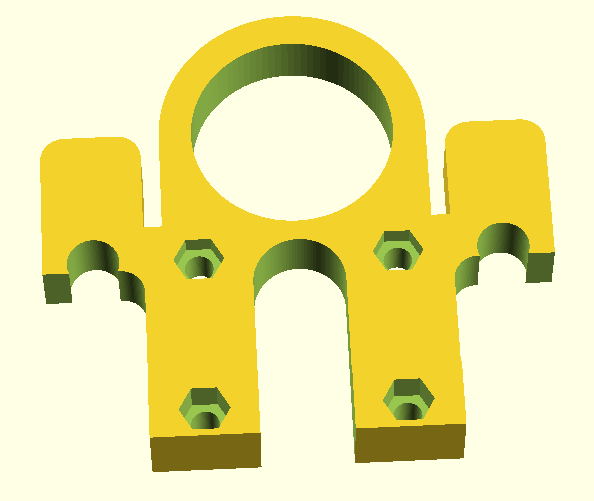

Supplement: File S1 — Figures S1–S10: 3-D printable parts for the open-source syringe pump. Figure S1 Carriage STL rendering. Figure S2 Carriage digital image. Figure S3 Clamp STL rendering. Figure S4 Clamp digital image. Figure S5 End idler STL rendering. Figure S6 End idler digital image. Figure S7 End motor STL rendering. Figure S8 End motor digital image. Figure S9 Wedges STL rendering. Figure S10 Wedges digital image. (ZIP) [file pone.0107216.s001.zip › Figure S3 - Clamp.tiff]

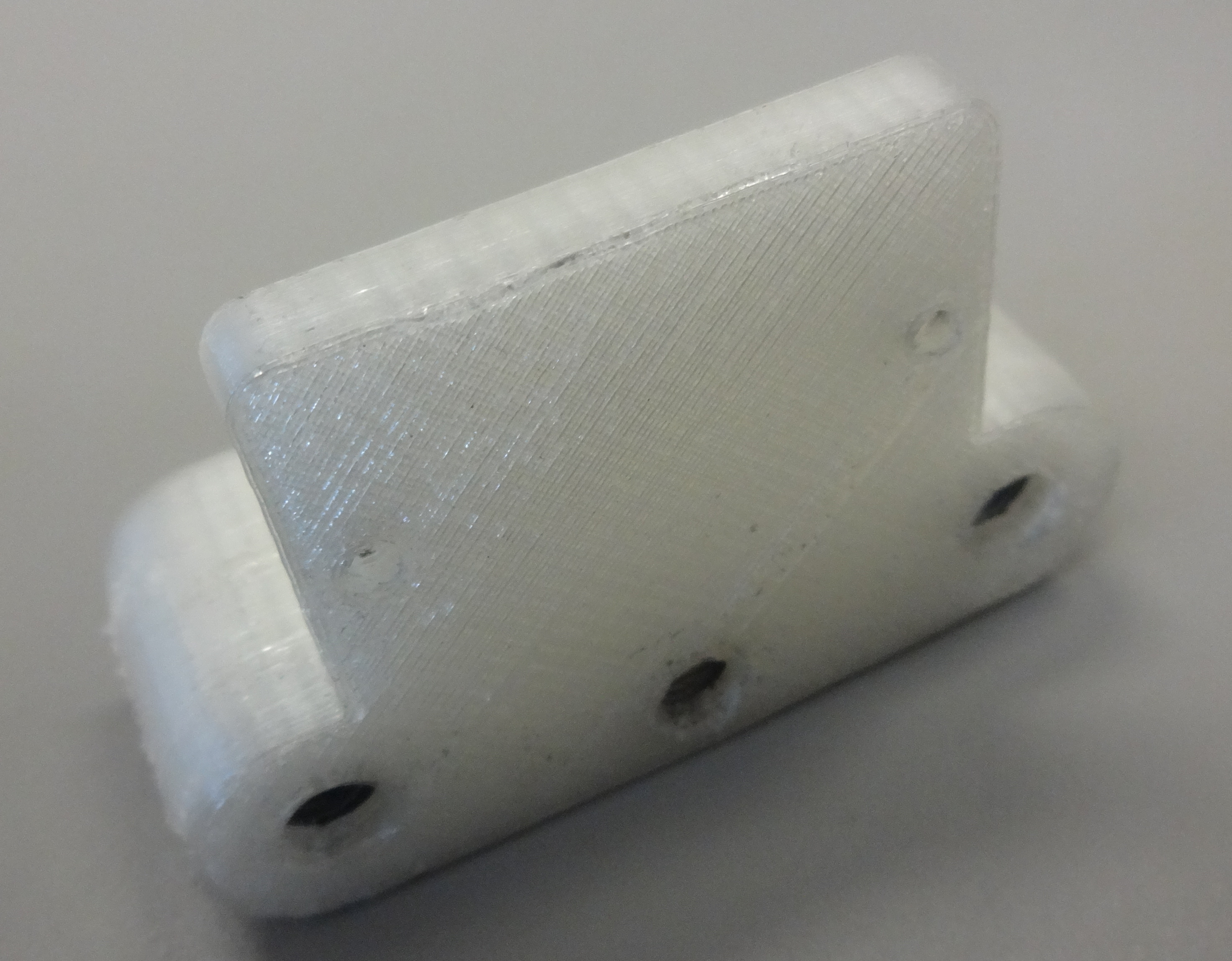

Supplement: File S1 — Figures S1–S10: 3-D printable parts for the open-source syringe pump. Figure S1 Carriage STL rendering. Figure S2 Carriage digital image. Figure S3 Clamp STL rendering. Figure S4 Clamp digital image. Figure S5 End idler STL rendering. Figure S6 End idler digital image. Figure S7 End motor STL rendering. Figure S8 End motor digital image. Figure S9 Wedges STL rendering. Figure S10 Wedges digital image. (ZIP) [file pone.0107216.s001.zip › Figure S2 -Carriage-p.tiff]

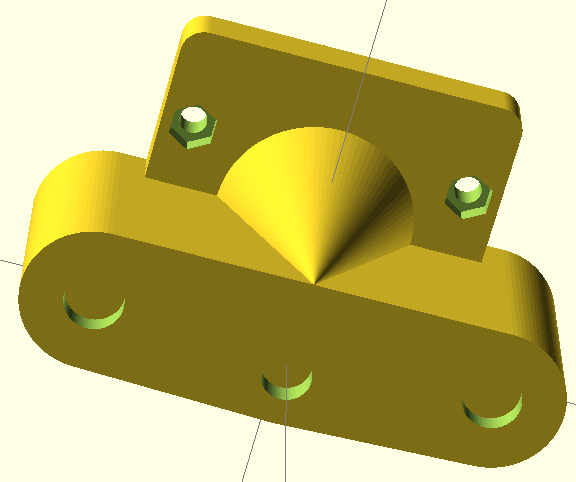

Supplement: File S1 — Figures S1–S10: 3-D printable parts for the open-source syringe pump. Figure S1 Carriage STL rendering. Figure S2 Carriage digital image. Figure S3 Clamp STL rendering. Figure S4 Clamp digital image. Figure S5 End idler STL rendering. Figure S6 End idler digital image. Figure S7 End motor STL rendering. Figure S8 End motor digital image. Figure S9 Wedges STL rendering. Figure S10 Wedges digital image. (ZIP) [file pone.0107216.s001.zip › Figure S1 - Carriage.tiff]
