# Supplementary material for: Open-Source Syringe Pump Library
Source: PLoS One. 2014 Sep 17;9(9):e107216. doi: 10.1371/journal.pone.0107216 (PMC4167991; doi:10.1371/journal.pone.0107216)
Supplement: Table S1 — Bill of Materials for three examples of the open-source syringe pumps. (DOC) [file pone.0107216.s002.doc]

| **Bill of Materials** | | | | | | |
| --- | --- | --- | --- | --- | --- | --- |
|  | **Nema 11** | | **Nema 17** | | **Dual Nema 17** | |
|  | **Part** | **Cost** | **Part** | **Cost** | **Part** | **Cost** |
| **Printed:** | Carriage, 20% fill | $1.59 | Carriage, 20% fill | $1.58 | Carriage x2, 20% fill | $3.15 |
|  | End: idler, 20% fill | $1.40 | End: idler, 20% fill | $1.88 | End: idler x2, 20% fill | $3.76 |
|  | Plunger Wedge, 20% fill | $0.60 | Plunger Wedge, 20% fill | $0.60 | Plunger Wedge x2, 20% fill | $1.21 |
|  | Syringe Clamp x2, 20% fill | $1.35 | Syringe Clamp x2, 20% fill | $3.27 | Syringe Clamp x2, 20% fill | $6.53 |
|  | End: Motor, 20% fill | $1.33 | End: Motor, 20% fill | $1.91 | End: Motor x2, 20% fill | $3.81 |
| **Hardware:** | Nema 11 motor | $15.95 | Nema 17 motor | $19.95 | Nema 17 motor x2 | $39.90 |
|  | M3x40 Bolt x4 | $0.35 | M3x40 Bolt x4 | $0.35 | M3x40 Bolt x8 | $0.70 |
|  | M3 Washer x8 | $0.14 | M3 Washer x8 | $0.14 | M3 Washer x16 | $0.28 |
|  | M3 Nut x10 | $0.10 | M3 Nut x10 | $0.10 | M3 Nut x20 | $0.21 |
|  | M5 Nut x5 | $0.09 | M5 Nut x5 | $0.09 | M5 Nut x10 | $0.17 |
|  | M3x12 Bolt x6 | $0.64 | M3x12 Bolt x6 | $0.64 | M3x12 Bolt x12 | $1.28 |
|  | M5 Threaded Rod(1m) | $5.03 | M5 Threaded Rod(1m) | $5.03 | M5 Threaded Rod(1m) x2 | $10.06 |
|  | Linear Ball Bearings | $4.66 | Linear Ball Bearing x2 | $4.66 | Linear Ball Bearing x4 | $9.32 |
|  | M3x16 Bolt x4 | $0.51 | M3x16 Bolt x4 | $0.51 | M3x16 Bolt x8 | $1.02 |
|  | Z Coupler | $3.49 | Z Coupler | $3.49 | Z Coupler x2 | $6.98 |
|  | Double Shielded Ball Bearing x2 | $1.50 | Double Shielded Ball Bearing x2 | $1.50 | Double Shielded Ball Bearing x4 | $3.00 |
|  | Metal Rod D6mm (3ft) | $11.03 | Metal Rod D6mm (3ft) | $11.03 | Metal Rod D6mm (3ft) x2 | $22.06 |
|  | Raspberry Pi | $40.00 | Raspberry Pi | $40.00 | Raspberry Pi | $40.00 |
|  | **Total** | **$89.76** | **Total** | **$96.72** | **Total** | **$153.44** |
